# Viral vectors for antimicrobial peptide expression: a new path for crop protection

**DOI:** 10.3389/fmicb.2026.1810690

**Published:** 2026-05-13

**Authors:** M. Basaloco, A. Albuquerque, J. A. Ribeiro, M. Patanita, F. Santos, T. Monteiro, M. D. Campos, C. Varanda, M. R. Félix

**Affiliations:** 1 MED Mediterranean Institute for Agriculture, Environment and Development & CHANGE Global-Change and Sustainability Institute, Institute for Advanced Studies and Research, Universidade de Évora, Évora, Portugal; 2 Research Centre for Natural Resources, Environment and Society (CERNAS), Santarém Polytechnic University, School of Agriculture, Santarém, Portugal; 3 Departamento de Fitotecnia, Escola de Ciências e Tecnologia, MED Mediterranean Institute for Agriculture, Environment and Development & CHANGE-Global Change and Sustainability Institute, Universidade de Évora, Évora, Portugal

**Keywords:** antimicrobial activity, biotechnology, plant protection, sustainable agriculture, transient expression systems

## Abstract

Antimicrobial peptides (AMPs) are key components of plant innate immunity, offering broad spectrum protection against pathogens and represent promising alternatives to chemical pesticides for sustainable crop protection. Despite their broad range antimicrobial activity and low potential for resistance development, the deployment of AMPs in agriculture has been severely limited by instability, poor bioavailability and the lack of efficient, field-compatible delivery strategies. Harnessing viral vectors as platforms for AMP expression in plants represents a powerful strategy to enhance plant innate immunity. This review provides an overview of the potential of viral vectors for transient gene expression, functional genomics and genome editing. We discuss the design, construction and delivery of viral vectors, as well as the main challenges associated with AMP expression, including cytotoxicity and stability. Finally, inspired by adeno-associated virus (AAV) mediated AMP delivery strategies in mammals, we propose a vaccine-like strategy for plant protection, in which viral vectors enable endogenous AMP production Although plants lack adaptive immunity, virus-mediated AMP expression may function as a biochemical analog, reinforcing basal defence layers and enhancing tolerance to pathogen infection. By integrating viral biotechnology with plant defence mechanisms, this approach could redefine the future of sustainable agriculture.

## 1 Introduction

Bacterial and fungal diseases are threatening agriculture in a global scale. More than 30% of crop yield losses are attributable to plant diseases, generating an economic burden on agriculture estimated at approximately 220 billion US dollars per year (Food and Agriculture Organization of the United Nations, 2025). Plant disease incidence has been rising in the past years, and it is predicted to follow the same tendency in the future (Kumar and Mukhopadhyay, 2025). Chemical pesticides, which are still being widely used besides restrictions, are very prone to induce microbial resistance due to its low specificity. In addition, antibiotics which have been under regulation review for the past years, and are forbidden in Europe for agricultural use, also present a high risk for the environment. Consequently, there is a pressing need to develop innovative and sustainable strategies to reduce reliance on chemical pesticides and antibiotics (Food and Agriculture Organization of the United Nations, 2021; Miller et al., 2022).

Among emerging strategies, antimicrobial peptides (AMPs) have gained attention due to their pivotal role on the innate immune system of living organisms by acting through immunomodulatory effects, microbial cell disruption, targeting microbial macromolecules, interacting with ATP-dependent enzymes or even by binding to nutrients. AMPs are small molecules, with molecular weights below 10 kDa, naturally found across animals, plants and microorganisms (Montesinos, 2007; Sun et al., 2025). By 1939, the first AMP, Gramicidin, was isolated from the supernatant culture of a soil *Bacillus* (Dubos, 1939). Their structure and biochemical nature are highly diverse, resulting in a variety of specificity and effectiveness. These bioactive molecules possess a large spectrum of antimicrobial activity, are safer for the environment and less susceptible to develop microbial resistance, posing a great advantage compared to traditional antibiotics (Sun et al., 2025; Wu et al., 2025). Therefore, AMPs have been widely used across several areas including medicine (David and Rajasekaran, 2015; Holaskova et al., 2015; Sharma et al., 2022; Zheng et al., 2025). Despite their great potential for plant disease management, its application on agriculture has not been fully explored due to stability, membrane permeability and bioavailability issues (Nazarian-Firouzabadi et al., 2024).

The transient expression of AMPs consists in the introduction of AMP encoding genes into living organisms without integrating the host’s genome. This methodology capacitates plants to rapidly produce high concentrations of AMPs with complex structures; a major advantage when compared to the traditional antimicrobial approaches. Vaccine development and gene therapy are some of the useful applications of this technology (Tang et al., 2023; Akher et al., 2025). To fully unlock the protective efficacy of these peptides in plant disease management, the development of innovative delivery systems is imperative. While biotechnological advances in plant molecular farming have enabled the use of plants as bioreactors for producing therapeutic AMPs for medicine, for instance, their application in enhancing plant defences against pathogens has received comparatively little attention (Chahardoli et al., 2018; Rutter et al., 2024; Akher et al., 2025). Although the antimicrobial potential of AMPs has been well documented, their practical use in crop protection remains limited, largely due to the lack of efficient, stable, and field-compatible delivery strategies. In this context, viral vectors emerge as powerful platforms. The ability of plant viruses to stably and asymptomatically deliver genetic material into host plants renders them highly effective vectors for the transient expression of AMPs (Gleba et al., 2007; Kujur et al., 2021; Akher et al., 2025). Therefore, due to viral vectors and *Agrobacterium* natural infective properties, biotechnology might evolve into combining both for the successful production of AMPs in plants. Plants expressing bioactive compounds, such as AMPs, to use for crop improvement represent a major biotechnological advance in agriculture (Breen et al., 2015).

The combined use of viral vectors and *Agrobacterium* infiltration for AMPs delivery and expression in plants is going to be addressed in this review, to highlight the potential of this biotechnological tool and providing new insights for plant disease management.

## 2 Antimicrobial peptides: a resource for plant defence

Plant innate immune responses generally start with stress recognition by specific receptors, triggering transduction cascades that generate the production of secondary metabolites and/or stress-related proteins (Campos et al., 2018). AMPs represent a group of these the stress-related proteins and are mainly produced as response to biotic stresses. This mechanism is not exclusive from plants, since AMPs can be found in all life domains from bacteria to fungi and even animals (Wu et al., 2025). Amphibians, for example, are one of the largest sources of AMPs, with more than 1,000 entries in the Antimicrobial Peptide Database (APD6) (Wang et al., 2026).

AMPs are naturally occurring small peptides, consisting of 5 to 100 amino acids, typically amphipathic and cationic. They can be synthesized in ribosomes, as the case of bacteriocins, or non-ribosomally produced, as is the case for the majority of bacterial proteins. These molecules exhibit antimicrobial activity against a broad range of targets, including viruses, protozoa, bacteria, fungi and even neoplastic cells (Kulaeva et al., 2020). Plant AMPs include defensins, snakins, thionins and cyclotides. Their effects are mediated through various mechanisms such as direct killing using membrane-disruptive mechanisms or using intracellular targeting such as binding to nutrients, targeting microbial macromolecules, inhibiting protein synthesis or constraining enzymatic activity. Moreover, AMPs can also act through immune modulation to enhance the organism’s immune system, by stimulating immune cell differentiation, chemotaxis or by interfering with cell wall synthesis (Bolouri Moghaddam et al., 2015; Farvardin et al., 2024; Jiang et al., 2025). Therefore, natural plant or fungal defensins, bacterial lantibiotics and bacteriocins, as well as synthetically produced peptides in *E. coli*, yeasts or plants such as tobacco, are few examples of the versatile AMP sources and platforms for its safe production (Cresti et al., 2024). There are three types of AMPs regarding their origin: natural, synthetic and hybrid. The natural AMPs are naturally produced by living organisms and are involved in innate immunity. The synthetic AMPs, are designed to reduce toxicity, improve stability and enhance specificity, enabling modifications for more targeted approaches (Montesinos and Bardají, 2008; Sun et al., 2025). For instance, a recent study revealed a synthetic designed AMP (APP3-14) that can act as a stabilizer of immune regulators, such as MYC2, an important transcription factor that takes part in the jasmonate signaling pathway, conferring citrus resistance to Huanglongbing, commonly known as citrus greening (Zhao et al., 2025). Moreover, the hybrid AMPs, which consist in the synergistic conjugation of AMPs with different modes of action for enhanced bactericidal activity or reduced toxicity (Mendes et al., 2021). Plant AMPs can function as damage-associated molecular patterns to activate defence responses, acting in pattern-triggered immunity but also in effector-triggered immunity, as shown in recent studies that highlighted the dual role of AMPs as antimicrobials and signaling molecules in plant systems (Bolouri Moghaddam et al., 2015; Farvardin et al., 2024; Jiang et al., 2025). Despite not conferring complete immunity, AMPs often act by reducing pathogen load, modulating disease symptoms and delaying disease progression, thereby enhancing plant tolerance and resilience to infection.

Several AMPs have been described as effective against many plant pathogens like *Fusarium* sp., *Phytophtora* sp., *Colletotrichum* sp., *Xanthomonas* sp.; *Verticillium* sp.; *Botrytis* sp., *Alternaria* sp. and *Magnaporthe* sp. (Tang et al., 2023; Kumar et al., 2024; Kalunke et al., 2025).

The foliar application of this type of compounds is commonly tested, such as the case of an AMP from *Lactiplantibacillus argentoratensis* that exhibited an inhibition capacity, in tomato plants, against *Ralstonia solanacearum*, the causal agent of bacterial wilt disease (Tiwari et al., 2024). Although foliar application seems to be a good alternative for disease management, AMPs stability and efficacy can be compromised due to the extreme exposure to enzymes, reducing their bioavailability and consequently effectiveness, resulting in the need for frequent re-applications (Jiang et al., 2025; Sun et al., 2025). To overcome this problem, the genetic engineering of plants encoding for AMPs, has started to be explored. Therefore, transgenic plants expressing AMPs will have a competitive advantage due to its constitutive or transient expression of antimicrobial peptides, ensuring a continuous protection against phytopathogens (Zhang et al., 2016; Li et al., 2021; Wu et al., 2025).

Transgenic plants that can express and accumulate high concentrations of AMPs represent an important tool for peptide production and extraction, as well as for direct agricultural application, as a disease management strategy. The use of tomato transgenic plants expressing AMPs coding genes was already reported to have improved resistance against *Clavibacter michiganensis* subsp. *michiganensis* and *Pseudomonas syringae* pv. *tomato* (Alan et al., 2004; Jan et al., 2010; Li et al., 2021). Some studies have also demonstrated cross-species functionality between different plant types, such as the case of some spinach defensins used in tomato and citrus (Padilla et al., 2025). In addition, the expression of the plant strong promoter pro-SmAMP2, from *Stellaria media*, in transgenic potato has shown enhanced resistance to the phytopathogens *Alternaria* sp. and *Fusarium* sp. (Komakhin et al., 2016). Furthermore in 2020, Niu et al. (2020) demonstrated that transgenic soybeans overexpressing CaAMP1 exhibited more tolerance to *Phytophthora sojae* (Niu et al., 2020).

There are plenty of advantages of using AMPs over traditional antibiotics, starting from the low probability of resistance development, the broad-spectrum of activity, their proven effectiveness in quorum sensing inhibition or in biofilm disruption. Also, with the biotechnological and bioengineering improvements, synthetic AMPs analogs have been developed to counteract toxicity issues as well as for enhancing yield and specificity. Moreover, the use of pants as production systems offers a scalable and cost-effective AMP synthesis, ensuring its correct post-translational modifications and thus conferring functionality (Chaudhary et al., 2023; Kawmudhi et al., 2025; Yang et al., 2025).

Despite their considerable potential, the practical application of AMPs in crop protection remains constrained by several factors, including susceptibility to proteolytic degradation, limited stability under field conditions and challenges associated with efficient delivery and expression in planta. While transgenic approaches have shown promise, they are often constrained by regulatory requirements and extended development timelines (Wu et al., 2025; Zheng et al., 2025).

These limitations underscore a central bottleneck in AMP-based strategies: the lack of efficient, scalable and flexible delivery systems. In this context, plant viral vectors have emerged as a compelling alternative, enabling rapid, high-level and systemic expression of AMPs in planta, thereby addressing key challenges associated with their deployment.

## 3 Viral vectors for gene expression in plants

To overcome the limitations associated with conventional AMP delivery strategies, plant viral vectors have emerged as highly effective alternative platforms for gene expression in plants. These systems enable systemic high-level and rapid production of recombinant proteins, being therefore particularly well suited for AMPs expression (Sainsbury and Lomonossoff, 2008; Saunders and Lomonossoff, 2013). By exploiting the natural infection and replication mechanisms of viruses, viral based vectors can facilitate efficient AMP accumulation without requiring stable genetic transformation, thus providing a flexible and scalable alternative to transgenic approaches.

Viral vectors are engineered plant viruses tailored to deliver genetic material into plant cells. The removal or silencing of genes related with viral pathogenicity, replacing them by genes of interest, is a common approach when using these systems (Varanda et al., 2018; Zaulda et al., 2022). Virus intrinsic nature to infect and replicate within plants represents a huge potential for biotechnological approaches, like the development of vaccines, production of recombinant proteins, gene expression and for exploring functional genomics (Akher et al., 2025).

Back in the 1980s, the first plant viral vectors were developed using *Cauliflower mosaic virus* (CaMV) and *Tobacco mosaic virus* (TMV), which enabled exogenous gene expression in plants (Howell et al., 1980; Donson et al., 1991). Since 2000, viral vectors have emerged as very important tools in plant molecular farming and functional genomics, as proven by the development of virus-induced gene silencing (VIGS). This tool exploits the plant’s natural RNA silencing-based immune defence against viruses, leading to targeted RNA degradation (Wang et al., 2020). In 2023, *Cassava common mosaic virus* (CsCMV) was edited to act as an overexpression vector for the delivery of single guide RNAs into Cas9-overexpressing cassava lines (Tuo et al., 2023). Also, the incorporation of CRISPR/Cas systems into viral platforms has enabled precise and efficient genome editing in a variety of plant species (Ribeiro et al., 2024). Employing viruses as tools for genome modification through virus-induced genome editing (VIGE) marks a major milestone in the evolution of modern biotechnology (Wang et al., 2020; Oh et al., 2021). More recent developments have shifted attention toward the refinement of viral vector properties, emphasizing advances in multiplexing and expression precision (Nagalakshmi et al., 2022; Ishibashi et al., 2024). New improvements include the design of deconstructed viral vectors, where non-essential components are removed to enhance biosafety, and synthetic biology-driven modifications for the optimization of expression and stability (Liu et al., 2023; Kim et al., 2024).

By enabling rapid systemic expression of genes, in high concentration levels, without necessarily needing a stable genomic integration, these vectors represent a very powerful and important biotechnological tool which complements stable transgenic approaches. This methodology also supports transient expression, enabling metabolic engineering, biopharmaceutical production, functional genomics and even crop trait improvement (Choi et al., 2019; Peyret et al., 2019; Sindarovska and Kuchuk, 2024).

As these platforms continue to expand their biotechnological relevance, new viral engineering strategies are pushing the boundaries of what can be achieved, enabling increasingly sophisticated applications ranging from multiplexed gene expression to next-generation silencing systems.

Recent studies have demonstrated simultaneous gene expression in maize, using *Sugarcane Mosaic Virus* (SCMV). The use of a potyvirus, was proven to be effective for dual heterologous gene expression in monocots, emphasizing once more its effectiveness in a variety of organisms and accentuating the capacity of multiplexing, even in challenging systems (Li et al., 2025). Also, a system denominated Virus-Mediated Short RNA Insertions (vsRNAi) was newly developed. The system takes advantage of viral characteristics to deliver very small RNA sequences that will further activate gene silencing. This strategy reduces construct complexity, improves scalability and broadens industrial applications (García et al., 2025). Furthermore, advances at the interface of computational and synthetic biology are expected to deliver next-generation viral vectors with superior precision and host flexibility, reinforcing their value in sustainable agriculture and molecular farming (Khakhar and Voytas, 2021).

Viral vectors can be divided into DNA or RNA vectors, depending on the nature of their nucleic acid genome, differing accordingly in the mechanism of action, stability and, consequently, applications, as represented in Table 1.

**Table 1:** Overview of widely used plant viruses employed as platforms for heterologous gene expression.

Viral backbone	Type	Replication Site	Applications	Host	Features	Limitations	References
*Tobacco mosaic virus* (TMV)	(+) ssRNA	Cytoplasm	Functional genomics	Solanaceae	Easy to engineer	Cargo capacity	Donson et al. (1991)
*Potato virus X* (PVX)	(+) ssRNA	Cytoplasm	Protein production	Solanaceae	Fusion proteins	Cargo capacity	Chapman et al. (1992)
*Tobacco rattle virus* (TRV)	(+) ssRNA	Cytoplasm	VIGS; Functional genomics	Solanaceae; other dicots	Gene silencing	Cargo capacity	Ratcliff et al. (2001)
*Cauliflower mosaic virus* (CaMV)	dsDNA	Nucleus	Stable and transient expression	Brassicaceae	Strong expression	Systemic spread; cargo capacity	Abrahamian et al. (2020) and Brisson et al. (1984)
Geminiviruses (BeYDV, WDV, MSV)	ssDNA	Nucleus	Genome editing	Monocots and dicots	Cargo capacity	Regulatory issues	Baltes et al. (2014) and Čermák et al. (2015)
*Foxtail mosaic virus* (FoMV)	(+) ssRNA	Cytoplasm	sgRNA delivery; Functional genomics	Monocots	Genome editing	Slow systemic spread	Bouton et al. (2018) and Liu et al. (2016)
*Cassava common mosaic virus* (CsCMV)	(+) ssRNA	Cytoplasm	sgRNA delivery; VIGE	*M. esculenta* and *N. benthamiana*	Overexpression	Limited host range	Tuo et al. (2021, 2023)
*Sugarcane mosaic virus* (SCMV)	(+) ssRNA	Cytoplasm	Multiplexing; Metabolic engineering	Monocots	Dual heterologous expression	Cargo capacity	Chung et al. (2021) and Mei et al. (2019)

DNA viral vectors are formed by a small single-stranded DNA molecule (ssDNA) which can carry a gene of interest. Geminiviruses are DNA-based viral vectors that belong to the most widely used plant virus family (Geminiviridae) for vector construction, amongst them: *Bean yellow dwarf virus* (BeYDV), *Wheat dwarf virus* (WDV), *Tomato golden mosaic virus* (TGMV) and *Maize streak virus* (MSV). This type of vectors can be applied for gene expression or silencing and replicate within the cell nucleus, achieving high copy number rates and thus leading to good high-yield expression. Their ability to target inaccessible tissues represents also an advantage of DNA based viral vectors over RNA viral vectors due to their persistence in the nucleus resulting in long-term gene expression (Kim et al., 2024; Zhang and Deng, 2026).

On the other hand, TMV, *Tobacco rattle virus* (TRV) or *Potato virus X* (PVX), are categorized as RNA viral vectors. Usually, RNA viral vectors replicate in the cell cytoplasm, avoiding genome integration, and thus are considered a fast and safer approach for transient expression, except for retroviruses, which integrate into the host genome. RNA viral vectors move systemically through the vascular system and plasmodesmata, enabling high protein expression across several tissues (Khakhar and Voytas, 2021; Uranga et al., 2023).

The use of viral vectors for AMP expression is particularly attractive given some related functional constraints such as the dependence on controlled expression levels to balance efficacy with potential phytotoxicity. Viral systems enable transient and tunable expression, thereby minimizing long-term metabolic burden on the host plant. In addition, their capacity for multiplexing allows the simultaneous expression of AMPs, which can expand antimicrobial activity and reduce the probability of resistance development.

Henceforth, viral vectors are highly versatile and powerful platforms for plant biotechnology, offering rapid, high-level gene expression, multiplexing capabilities, and the potential to accelerate functional genomics, molecular farming, and crop improvement (Kujur et al., 2021). Accordingly, viral vector systems can be viewed not merely as expression tools, but as enabling technologies for the practical implementation of AMP-based plant protection strategies. However, realizing their full potential requires careful vector design to ensure stability, safety, and host compatibility, alongside with rigorous evaluation of their field applicability. Striking this balance between innovation and diligence will be essential to harness viral vectors as reliable and scalable tools for sustainable agricultural applications.

### 3.1 Delivery methodology

Depending on the plant species, virus type and final objective, if it is for VIGS, CRISPR/Cas delivery, functional genomics or transient expression, the delivery method applied to the host must vary accordingly.

There are several methods for viral vector delivery into plants, such as mechanical inoculation, agroinfiltration, particle bombardment, protoplast transfection and by biological carriers, like insects, including aphids and whiteflies. In this review we will focus on the most used methodologies: mechanical inoculation, agroinfiltration and particle bombardment (Chen and Lai, 2015; Monroy-Borrego and Steinmetz, 2022).

Mechanical inoculation has been used for more than a century, with early demonstrations dating back to 1892, and has become a standard technique in plant virology by the 1920s due to its simplicity (Scholthof et al., 2022). Despite its early discovery and its traditional use for the transmission of RNA viruses, this methodology was only applied in biotechnology for *in vitro*-transcribed RNA viral vectors later, in the 1980s (Lico et al., 2008). The technique involves the physical introduction of viral particles into plant cells through microscopic leaf wounds produced by abrasive agents such as carborundum or celite. Inoculation can be performed using sap from infected to healthy plants, by directly applying a transcribed RNA viral genome under a strong promoter onto the leaves or through pressurized spray inoculation (Hull, 2009). The method has been reportedly proven effective across a variety of hosts, spanning from monocots to dicots and can be used in multiple applications, including VIGS, transient expression or pathogenicity testing. However, its applicability to DNA viral vectors is limited, as many DNA viruses require nuclear import and access to host transcription machinery, provided by alternative delivery systems. Despite this limitation, mechanical inoculation offers distinct advantages, including the rapid onset of symptoms, procedural simplicity, cost-effectiveness, the capacity for direct RNA particles delivery and the possibility to be applied for many viruses. Nonetheless, certain drawbacks must be acknowledged, including its low efficiency in some waxy-leaf species such as cereals, the impossibility of upscaling the method, tissue stress and damage, variable infection rates and the requirement of viable virions or RNA, which can be often challenging to ensure due to their susceptibility to degradation (Monroy-Borrego and Steinmetz, 2022).

Agroinfiltration or *Agrobacterium*-mediated delivery was first demonstrated in 1986 and exploits the intrinsic nature of *Agrobacterium tumefaciens* to transfer T-DNA into the plant cell nucleus without the need for tissue culture (Hille et al., 1986). This transient gene delivery methodology can be used for both RNA and DNA viral vectors. For RNA viruses, infiltration with a transformed *Agrobacterium tumefaciens* carrying an engineered binary vector containing the modified viral cDNA clone allows transcription of the viral RNA from a strong plant promoter, usually CaMV 35S, by the plant’s RNA polymerase. For DNA viruses, the binary vector delivers a DNA viral replicon, which is replicated in the nucleus and transcribed to produce viral RNA (Lindbo, 2007a).

In both cases, infiltration is performed using a syringe needle or a vacuum chamber targeting leaf intercellular spaces. The viral genome will then be replicated, and the gene of interest is expressed and potentially spread systemically. Transient gene expression may last for several days to weeks, not integrating into the host’s genome, until tissue senescence or host silencing mechanisms initiates, reducing expression (Lindbo, 2007a). Although agroinfiltration involves some regulatory considerations for using a genetically modified bacterium, this technique is widely employed due to its efficiency, flexibility, and scalability. The technique is usually applied for transient protein production (e.g., antibodies, enzymes, vaccines), functional genomics and protein interaction studies, VIGS and for the delivery of CRISPR/Cas components (Spiegel et al., 2022). Besides all the advantages of using agroinfiltration, its efficacy varies between plant species, tending to work better in *Nicotiana benthamiana* and other dicots, contrarily to monocots which remain a challenge (Zhang et al., 2020). Likewise, gene silencing mechanisms may also limit expression over time (Jay et al., 2023).

Finally, particle bombardment was first demonstrated in plants by 1987 and consists in integrating nucleic acids through microscopic metal particle bombardment onto plant cells or tissues (Klein et al., 1987). Also referred to as biolistics, this delivery method primarily used for gene transfer, employs tungsten or gold microparticles coated with viral genomes, which are accelerated at high velocity into plant tissues, enabling genomic material to enter the cell and, possibly, be expressed and replicated (Lacroix and Citovsky, 2020). In 1993, its application for geminiviruses inoculation in plants was one of the earliest reported uses as a viral delivery system (Garzon-Tiznado et al., 1993). Biolistics is characterized by its simple and quick methodology, allowing the delivery of infectious viral DNA or RNA clones and it has been explored for gene expression and silencing in recalcitrant tissues and species. Its efficacy varies depending on the targeted tissue, type of virus and even amongst plant species (Yamagishi and Yoshikawa, 2013; Thorpe et al., 2025). Furthermore, the high costs associated with the low efficiency when compared with other inoculation methods, the delayed appearance of symptoms alongside with the randomized nature of the technique represent some drawbacks of particle bombardment (Lacroix and Citovsky, 2020).

### 3.2 Application

The intended end use also plays a major role in the selection of viral vectors. Depending on the goal, the approach should be planned considering virus type, construct design and delivery methodology.

Viral vectors can be used in functional genomics through VIGS to study gene function, genome editing for the transient delivery of CRISPR-Cas components, molecular farming to produce recombinant proteins and in synthetic biology for modulation of biosynthetic pathways.

In the 1990s, transient gene expression was one of the first reported viral vector applications using TMV and PVX in *Nicotiana* spp. (Garzon-Tiznado et al., 1993; Kumagai et al., 1995). The quick expression of foreign genes in plants, without altering permanently the host’s genome allowed to produce enzymes, antibodies or even vaccines and to test gene function prior to a stable transformation (Sindarovska and Kuchuk, 2024). This technique is highly efficient when the aim is to produce target proteins without stable integration, and it is widely used in industrial molecular farming, where plants serve as bioreactors to produce antibodies and vaccines, like the Medicago’s CoVLP vaccine for COVID-19 (Ward et al., 2021) or the ZMapp for Ebola (Qiu et al., 2014), both manufactured in *Nicotiana benthamiana*. Molecular farming holds a high biotechnological and commercial potential since it combines high yields, easy scalability and low production costs. Based on its principles, a wide range of plant biotechnology approaches have since been developed, all derived from designing vectors to temporarily deliver and express target genes for diverse applications (Akher et al., 2025). Transient expression and its variants represent highly versatile strategies since they can be applied utilizing a variety of vectors (Shah et al., 2013).

Still in the 1990s, VIGS, a very useful method for functional genomics was developed (Ruiz et al., 1998). By observing the effects of knocking down or silencing specific genes, this technique can also be applied for studying plant immunity and signaling defence mechanisms (Deng et al., 2025). The use of VIGS to uncover resistance or susceptibility related genes became very popular worldwide, particularly using TRV, Barley stripe mosaic virus (BSMV) and Foxtail mosaic virus (FoMV) (Huang, 2017; Beernink and Whitham, 2023; Deng et al., 2025). The technique exploits the plant’s RNA silencing machinery, whereby a host-gene fragment inserted into the viral vector elicits production of small interfering RNAs that consequently direct the degradation of the corresponding endogenous mRNA (Velásquez et al., 2009). Additionally, VIGS can also be adapted for transient gene expression (Ogata et al., 2021). This system is mostly used to investigate plant defence responses against biotic stresses, however, its broader application is constrained by the limited availability and host-range specificity of suitable viral vectors (Shingaliev et al., 2025).

Other approach that became widely adopted in plant biotechnology was VIGE, in which viral vectors are used to deliver CRISPR guided RNAs to plants expressing Cas9 (Tuo et al., 2023; Uranga et al., 2024). This strategy, similar to transient expression and VIGS, allows rapid gene modification without the need for stable transformation, which can be time-consuming and labor-intensive (Lee et al., 2024). VIGE systems can employ several viruses to be used as vectors for carrying and deliver the guide RNAs efficiently. The major limitation of this strategy directly relates with the need of transformed Cas9 plants as hosts, limiting the flexibility of the methodology (Mikhaylova, 2025).

More recently, the temporary introduction of regulators or enzymes to adjust or modify metabolic pathways, thereby enhancing the production of desired metabolites has attracted increasing attention among plant biotechnologists. This process, named metabolic reprogramming or metabolic pathway engineering, often uses synthetic biology approaches and involves the co-infection of plants with multiple viral vectors carrying different pathway-related genes (Majer et al., 2017). This strategy is particularly suitable for rapidly testing complex metabolic pathways. In plants, viral vector mediated metabolic engineering has been used to enhance valuable metabolites. For example, the introduction of phytoene synthase and lycopene *β*-cyclase into tobacco plants has been used to increase carotenoid accumulation (Majer et al., 2017) while the overexpression of flavonol synthase and chalcone isomerase in tomato has been shown to boost flavonoid content, thereby enhancing antioxidant properties (Muir et al., 2001).

Within these applications, AMP expression represents a particularly promising strategy that combines features of transient protein production and crop protection. Viral-vector mediated AMP expression can be viewed as a specialized case for molecular farming, where the produced peptides act directly within the plant to confer protecting against pathogens.

All these applications are dependent on the efficient, reliable and fast introduction of genetic material into plant cells, making the choice of the vector system a critical determinant of their success.

### 3.3 Design and construction

Selection of the viral backbone should be based on the host range, expression characteristics and genomic organization of the virus. For construct design, the gene of interest should be under the influence of a strong promoter, like CaMV 35S or another suitable plant or viral promoter (Mahmood et al., 2023). In RNA viral vectors, the expression cassette may be inserted downstream of a subgenomic promoter or replace a non-essential viral gene, to ensure transcription, whereas in DNA viral vectors it is commonly incorporated as a transcriptional unit within the replicon (Kim et al., 2024). Under specific circumstances, to ensure systemic spread, certain viral genes, such as those encoding for coat or viral proteins may be retained to enable systemic movement (Akher et al., 2025). The inserted sequence should be codon-optimized for the host, stabilized to minimize recombination and might include fusion tags (e.g., His-tag, GFP) to enable easy detection and purification of the expressed protein or peptide (Coates et al., 2022; Jung et al., 2025). DNA viral vectors already exist in the form of replicons, thereby facilitating manipulation contrarily to RNA viral vectors that mimic mRNA expression systems, which require transcription of a cDNA copy to start replication (Zhang and Deng, 2026). Moreover, the two types serve for different purposes: DNA-based viral vectors, like BeYDV, are utilized for a more basal sustained expression with reduced cytotoxic effects (Diamos and Mason, 2019), while RNA viral vectors, such as *Tobacco mosaic virus* (TMV), can be used to test the efficacy of expression *in planta* relying on a rapid, high-level transient expression (Lindbo, 2007a,b).

For AMP expression, construct design must also be considered to overcome peptide related constrains. Those include potential cytotoxicity, proper folding and stability, which may require the use of targeting signals or controlled expression systems.

## 4 RNA-based viral vectors: a versatile platform for gene delivery

The natural ability of viruses to infect and replicate within plant cells makes them excellent candidates to be used as vectors in gene delivery. Although both DNA and RNA viral vectors might be employed for the aforementioned purposes, RNA plant viruses have particularly interesting characteristics that are advantageous for a quick high-level protein delivery and expression. As previously noted, these viruses replicate autonomously in the cytoplasm, thereby avoiding genomic integration and allowing rapid, transient, and high-level expression without the risk of heritable transmission (Lindbo, 2007b). Moreover, RNA viruses spread systemically through vascular tissues and plasmodesmata, enabling whole-plant delivery of the introduced gene of interest (Kappagantu et al., 2020; Khakhar and Voytas, 2021). These features make RNA viral vectors particularly well suited for the transient and high-throughput applications highlighted in the previous section.

Plant RNA viral vectors are derived from modified RNA plant viruses and serve as efficient platforms for the expression and delivery of exogenous genes in plant hosts (Akher et al., 2025). Simplicity and speed are two of the emphasized features of this delivery system (Achs et al., 2024). Such vectors can be employed for the expression of proteins or peptides, for VIGS, or for the delivery of CRISPR/Cas machinery, thereby demonstrating high versatility in plant biotechnology applications (Lou et al., 2024).

The majority of plant viral vectors are derived from positive-sense single stranded RNA ((+)ssRNA) such as TMV, PVX, BSMV and *Cowpea mosaic virus* (CPMV). (+)ssRNA viral vectors act directly as mRNA once in the cytoplasm, making them particularly suitable for protein expression (Reddy et al., 2025). After entering the plant, typically through Agrobacterium-mediated delivery or mechanical inoculation, the viral RNA is recognized by the plant’s translational machinery, allowing rapid production of viral replicase proteins required for genome amplification. These proteins synthesize complementary negative-sense RNA strands, serving as template for generating large amounts of new (+)ssRNA. The constitutive high-level transcription of the introduced foreign genes and following expression of recombinant proteins or regulatory elements is ensured by the viral subgenomic strong promoters (Shen et al., 2024). Because (+)ssRNA viruses replicate exclusively in the cytoplasm, expression occurs extremely quick, within few days. Also, the native ability to move from cell-to-cell through plasmodesmata and systemically through phloem allows expression across large regions of the plant (Zhang et al., 2025).

Due to their robust replication and strong subgenomic promoters, TMV-based vectors are widely used for high-yield transient expression. Usually, this virus naturally infects a broad range of solanaceous hosts, spreads rapidly and systemically and tolerates the insertion of relatively large genomic sequences ensuring stability (Huang et al., 2024). When the primary objective is rapid expression and vigorous heterologous protein production, TMV-derived systems are the ones typically adopted.

Moreover, TRV-based vectors have become the predominant system for VIGS due to their ability to infect meristematic tissues, to trigger potent RNA interference responses and for causing mild symptomatology (Li et al., 2024). These viruses establish systemic but low-symptomatic infection, allowing the plant to remain viable while maintaining effective silencing signals. This system is therefore highly useful for functional genomic applications requiring stable, systemic gene silencing (Deng et al., 2025).

Lately, the development of hybrid systems combining elements from different viruses to optimize host range and expression levels has become very popular. In some cases, taking advantage of viral replication machinery while removing nonessential viral genes such as movement proteins, has also been explored to retain infection, avoiding biosafety issues (Jung et al., 2025).

The rapid high-level expression, cytoplasmic replication combined with an efficient systemic spread, make (+)ssRNA viral vectors one of the most widely used and powerful platforms for transient gene expression and its many derivatives, either to functional genomics or genome editing (Shen et al., 2024).

## 5 Limitations and opportunities for RNA viral platforms

### 5.1 Biological and molecular constraints

Although viral vectors represent highly versatile platforms for transient expression, especially RNA-based viral vectors that offer powerful means for all the applications, their deployment is accompanied by several inherent limitations and practical constraints that should be considered. One of the most critical constraints is the insert size since RNA viruses have a limited genomic cargo capacity typically ranging from 0.5 kb to 1.0 kb. This severely limits the expression of some proteins, fusion constructs or even multiplexed designs that require multiple genetic elements. In addition, the high viral recombination rates can decrease insert stability, reduce expression duration and interfere with downstream functional assays (Avesani et al., 2007; Shen et al., 2024).

Viral replication can also trigger plant immune responses, including RNA silencing, which can supress viral activity and consequently reduce transgene retention (Lopez-Gomollon and Baulcombe, 2022).

### 5.2 Host-range restrictions

Most available viral vectors exhibit narrow host ranges, thus limiting applicability to a small number of model species or some annual crops (Tuo et al., 2023). Monocots, woody perennials and fruit trees remain particularly underserved due to the absence of efficient, well-characterized viral-based vector systems. As many of these species represent economically important crops and are mainly recalcitrant to stable transformation, so there is a bottleneck that should be addressed (Zang et al., 2020). Their long life cycles, propagation methods and complex tissue organization make these species ideal candidates for transient expression strategies. However, the lack if suitable viral vectors hinder the ability to test some proteins and peptides directly in relevant host-pathogen contexts across several crop groups (Cui and Wang, 2016).

### 5.3 Ecological considerations

To prevent unintentional spread, cross-contaminations or environmental release, the development and use of viral vectors require a careful biosafety management (Brewer et al., 2018). Containment can be particularly challenging for systemic spreading vectors or vectors that can be mechanically transmitted (Hefferon, 2012). Additionally, viral infection and high-level transgene expression may cause physiological burdens to the host plant including metabolic shifts or growth retardation (Casteel and Falk, 2016).

### 5.4 Regulation and public perception

The implementation of genetically modified organisms introduces further complexity. In the European Union, stringent regulations and oversight have heightened public scepticism toward transgenic approaches. These regulatory frameworks limit field and industrial applications and consequently the commercialization of viral vector systems (European Parliamentary Research Service, 2023; Council of the European Union and European Parliament, 2025). Even when solely used for research, extensive containment and compliance requirements can restrict experimentation and slow the adoption of innovative viral technologies for plant protection studies (Zimny, 2023; Council of the European Union, 2025).

In contrast, regulatory approaches in Asia and the Americas are more heterogeneous and, in some cases, more permissive. In the United States, risk based regulatory frameworks emphasize the characteristic final product rather than the genetic modification process, facilitation field trials and commercialization (National Academies of Sciences, Engineering, and Medicine, 2016). Also, China and Japan have been investing in agricultural biotechnology with regulatory systems that allow controlled field experimentation following approval (Ishii and Araki, 2017). Collectively, these approaches may enable a faster translation of viral vector technologies into practical applications compared with the more precautionary European framework.

### 5.5 Limitations on the use of viral vectors for AMP expression

Although plant viral vectors have been used to express a wide range of heterologous proteins successfully, and AMPs have been extensively studied through stable transgenic approaches, their combined use remains limited. Few number of studies have explored AMPs expression directly through viral vectors (Nazarian-Firouzabadi et al., 2024). This gap persists partly due to some AMPs associated cytotoxicity, interference with systemic movement and, sometimes, the requirement of post-translational modifications, secretion pathways or proteolytic processing to ensure functionality (Hoelscher et al., 2022; Sun et al., 2025). As a result, stable transformation systems have been favored over transient viral approaches, despite their longer development timelines and stricter regulatory constraints.

One of the most significant constraints in AMP expression using viral vectors is cytotoxicity. Due to their membrane-disruptive properties, AMPs can impair host cell integrity when in high intracellular concentrations, triggering stress responses or localized tissue damage (Breen et al., 2015; Campos et al., 2018; Wu et al., 2025). This issue is particularly pronounced in viral systems, which are typically optimized for rapid and high-level protein expression (Gleba et al., 2007; Lindbo, 2007a,b; Diamos and Mason, 2019). To mitigate these effects, several strategies have been explored, including optimization of expression levels, subcellular targeting to reduce unintended interactions with essential cellular components, and the use of fusion constructs to stabilize peptides during accumulation (Holaskova et al., 2015; Coates et al., 2022; Chaudhary et al., 2023). These approaches aim to balance antimicrobial efficacy with host compatibility while maintaining sufficient expression levels for biological activity.

Other important limitation relates to the requirement of post-translational modifications for the activity of many AMPs. Peptide maturation can be essential for stability and function but may not be efficiently supported in all viral expression contexts, particularly when peptides accumulate in the cytosol. Plant based expression studies have demonstrated that appropriate processing and folding are critical for AMP functionality, and that expression platform and cellular context play an important role in determining peptide activity (Holaskova et al., 2015; Chaudhary et al., 2023). Therefore, improving compatibility between AMP structural requirements and the expression environment remains a crucial consideration for successful implementation.

In addition, vector-related limitations such as insert size restrictions, generic instability and potential interference with viral replication or systemic movement can further impact AMP expression efficiency (Avesani et al., 2007; Abrahamian et al., 2020; Kappagantu et al., 2020). Ongoing advances in viral vector engineering, including the development of deconstructed systems and improved regulatory elements are helping to address some of these challenges by enhancing transgene stability and allowing more precise control of expression (Pasin et al., 2024; Akher et al., 2025).

These limitations underscore a clear necessity for developing new viral-based vector systems that provide broader host coverage, enhanced stability and improved compatibility with AMP expression. However, many of these challenges appear to be technical rather than fundamental, reflecting the need for improved integration between peptide design and expression platform features.

Overcoming these challenges would not only accelerate AMP screening under physiologically relevant conditions but also expand biocontrol innovation beyond model plants, unlocking the full potential of viral vectors and providing new pathways for sustainable crop protection in high-value agricultural sectors (Pasin et al., 2024; Akher et al., 2025).

Importantly, these constraints should not be viewed solely as barriers, but rather as guiding lines for the development of more refined application strategies. In particular cytotoxicity, peptide maturation and expression control related challenges suggest that constitutive, high level AMP production may not always represent the most effective or biologically compatible approach. Instead, alternative paradigms based on controlled, context-dependent or prophylactic expression may offer a more viable path forward. In this context, viral vectors can be re-envisioned not only as expression tools but as transient delivery systems for protective traits.

## 6 Future directions

Viral-vector mediated systems provide a practical platform for transient and targeted AMP expression in plants, leveraging their inherent capacity for rapid and systemic protein production. This approach supports the flexibility of the tools for enhancing crop protection while reducing reliance on conventional chemical substances.

In this regard, the viral-mediated transient delivery of AMPs as a form of plant protection strategy can be further explored. By enabling rapid, systemic and controllable expression of AMPs, these platforms may overcome several limitations associated with direct peptide application such as instability and rapid degradation, as well as the time constraints linked to stable transgenic approaches. The recent proposal of using viral-based vectors to deliver AMP genes in mammals offers a compelling conceptual framework that may be extendable to plant biotechnology. The underlying principles of AAV-mediated AMP delivery such as its compact size, high expression efficiency and the endogenous production of protective peptides can easily be translated to agricultural settings through the application of plant viral-based vectors. A new study has demonstrated that AAV-based viral vectors can provide sustained, and prophylactic AMP expression in mammals, circumventing limitations associated with direct peptide application like instability and rapid degradation (Baindara et al., 2025).

Similarly, plant systems offer analogs opportunities, since many plant viruses have already served as robust expression platforms for the quick dissemination of small genes throughout host tissues. Likewise, these vectors might also be adapted to deliver AMP genes into economically important crops, enabling *in situ* production of AMPs, thus enhancing resilience against pathogenic bacteria, fungi or oomycetes. This extension represents a natural conceptual extrapolation of the principles demonstrated in mammal systems in which endogenous AMP production may offer a broad-spectrum, durable and resistance-limiting form of protection compared to the conventional pesticides or antibiotics. Moreover, plant viral expression systems are characterized by rapid deployment and easy modularity, suggesting the possibility of tailoring AMP assemblies to specific pathogen threats, hosts or even environmental contexts (Akher et al., 2025; Neubauer et al., 2025). This flexibility further supports their use as adaptable tools in precision crop protection strategies.

**Figure 1:**
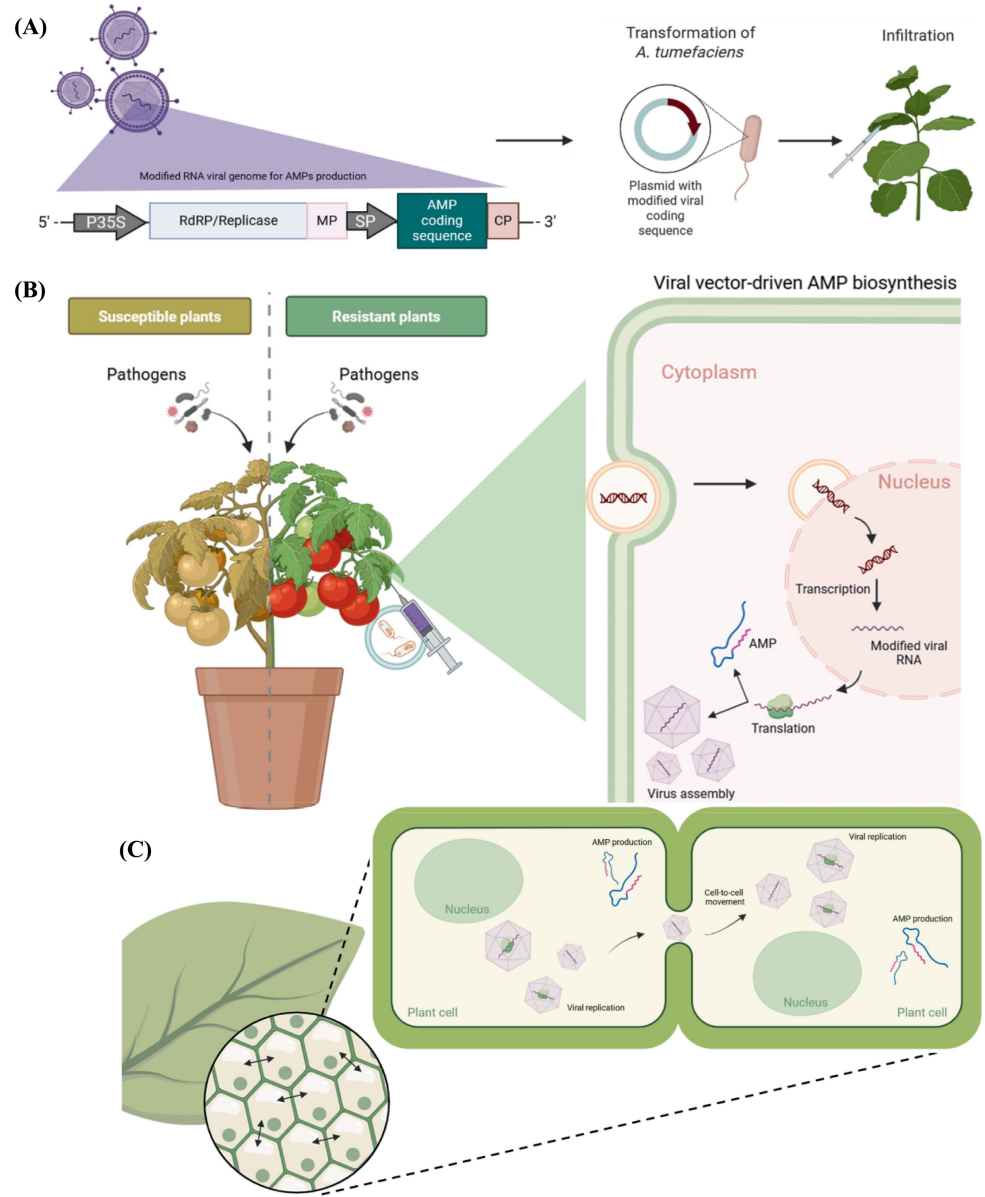
Schematic illustration of a vaccine-like approach as form of crop protection, using a plant viral vector for AMP delivery. **(A)** Viral vector design carrying a desired AMP coding sequence downstream of a strong promoter and *Agrobacterium* transformation followed by infiltration as delivery methodology. **(B)** AMP biosynthesis and cytoplasmic viral replication in a plant cell agroinfiltrated with a modified viral molecule carrying an AMP coding sequence. **(C)** Intercellular viral spread leading to AMP transient expression and production throughout the whole plant and consequent increased protection against pathogens. This figure was generated using a free version of BioRender.

The *vaccine-like*, prophylactic paradigm highlighted in the animal-focused review is also aligned with this suggested approach, schematically represented in Figure 1, where continuous or inducible transgene expression could avoid initial pathogen establishment, representing a prevention strategy for pathogenic control (Baindara et al., 2025). While plants lack adaptative immunity (Jones and Dangl, 2006), virus-based induced AMP expression could serve as a functional analog by establishing a persistent biochemical barrier against invasion (Liu et al., 2025).

However, several considerations such as optimal expression levels, tissue specificity, interactions with the plant microbiome, and potential fitness trade-offs, remain crucial areas for future investigation. Moreover, the biosafety and ecological implications of deploying AMP-expressing viral vectors in agricultural environments would require careful evaluation. Nonetheless, the cross-domain logic is compelling: if viral-mediated AMP delivery can enhance antimicrobial defences in animals, analogs plant viral systems may hold promise as innovative versatile and environmentally sustainable tools for an integrated next-generation crop protection strategy.

## 7 Conclusion

Viral-vector mediated expression of AMPs represents a versatile and sustainable strategy for plant protection. In the context of increasing global pressure to reduce chemical usage in agriculture and address the emergence of resistant plant pathogens, this approach offers a promising alternative on AMP expression and production *in planta*. Viral vectors offer rapid, systemic and high yield AMP production, providing broad spectrum antimicrobial activity with reduced associated resistance risk compared to conventional pesticides. AMP *in situ* expression could enhance crop resilience while minimizing chemical usage and thus allowing targeted responses to specific pathogens.

Overall, despite remaining technical and biological challenges, these systems also create opportunities for interdisciplinary research focused at overcoming current bottlenecks in AMP delivery and expression. Advances in viral vector engineering, synthetic biology and peptide design will improve applicability and performance in agricultural systems. Therefore, the use of viral vectors for AMP expression and delivery in plants constitutes a forward-looking platform for next generation crop protection, combining efficacy, flexibility and environmental sustainability.

## Glossary

(+)ssRNA: Positive-sense single-stranded RNA
AAV: Adeno-Associated Virus
AMP: Antimicrobial peptides
BvYDV: *Barley yellow dwarf virus*
BSMV: *Barley stripe mosaic virus*
CaMV: *Cauliflower mosaic virus*
Cas9: CRISPR-associated protein 9
cDNA: Complementary DNA
CPMV: *Cowpea mosaic virus*
CRISPR: Clustered Regularly Interspaced Short Palindromic Repeats
CSCMV: *Cassava common mosaic virus*
FoMV: *Foxtail mosaic virus*
GFP: Green fluorescent protein
His-tag: Polyhistidine tag
mRNA: Messenger RNA
MSV: *Maize streak vírus*
PVX: *Potato vírus X*
SCMV: *Sugarcane mosaic virus*
ssDNA: Single-stranded DNA
T-DNA: Transfer DNA
TGMV: *Tomato golden mosaic virus*
TMV: Tobacco mosaic virus
TRV: *Tobacco rattle vírus*
VIGE: Virus-induced genome editing
VIGS: Virus-induced gene silencing
vsRNAi: Virus-derived small interfering RNA
WDV: *Wheat dwarf virus*
